# Rapid response of osmotic stress transcription factor 1 (OSTF1) expression to salinity challenge in gills of marine euryhaline milkfish (*Chanos chanos*)

**DOI:** 10.1371/journal.pone.0271029

**Published:** 2022-07-06

**Authors:** Yu-Ting Lin, Tsung-Han Lee

**Affiliations:** 1 Department of Life Sciences, National Chung Hsing University, Taichung, Taiwan; 2 The iEGG and Animal Biotechnology Center, National Chung Hsing University, Taichung, Taiwan; National Cheng Kung University, TAIWAN

## Abstract

Euryhaline teleosts can survive in environments with different salinities. Cortisol is an important hormone for acclimation to seawater (SW) of euryhaline teleosts. Osmotic stress transcription factor 1 (OSTF1), also called the transforming growth factor-beta stimulated clone 22 domain 3 (tsc22d3), was first reported in tilapia as an acute response gene and protein under hyperosmotic stress, and it is regulated by cortisol. To date, most studies on OSTF1 have focused on freshwater inhabitants, such as tilapia, medaka, and catadromous eel. The expression of OSTF1 and the correlation between OSTF1 and cortisol in marine inhabitant euryhaline teleosts, to our knowledge, remain unclear. This study reveals the changes in the expression levels of branchial OSTF1, plasma cortisol levels, and their correlation in the marine inhabitant milkfish with ambient salinities. The two sequences of milkfish TSC22D3 transcripts were classified as OSTF1a and OSTF1b. Both genes were expressed universally in all detected organs and tissues but were the most abundant in the liver. Similar gene expression levels of *ostf1a* and *ostf1b* were found in SW- and fresh water (FW)-acclimated milkfish gills, an important osmoregulatory organ. Within 12 hours of being transferred from FW to SW, the gene expression level of *ostf1b* increased significantly (4 folds) within 12 h, whereas the expression level of *ostf1a* remained constant. Moreover, cortisol levels increased rapidly after being transferred to a hyperosmotic environment. After an intraperitoneal injection of cortisol, the gene expression levels of *ostf1a* and *ostf1b* were elevated. However, under hyperosmotic stress, *ostf1a* gene expression remained stable. Overall, the results revealed that *ostf1b* was the primary gene in milkfish responding to hypertonic stress, and cortisol concentration increased after the transfer of milkfish from FW to SW. Furthermore, cortisol injection increased the expression of *ostf1a* and *ostf1b*. As a result, factors other than cortisol may activate *ostf1b* in milkfish gills in response to an environmental salinity challenge.

## Introduction

Euryhaline teleosts can adapt to fluctuations in environmental salinity. In 2005, the osmotic stress transcription factor 1 (OSTF1) was first identified in the primary culture of gill epithelial cells of the Mozambique tilapia (*Oreochromis mossambicus*) upon hyperosmotic stress [1]. It is thought to be an early and transiently upregulated gene and protein in fresh water (FW) inhabitant euryhaline teleosts [1–3]. Further studies on Japanese medaka (*Oryzias latipes*) revealed two transcripts of *tsc22d3*. *ostf1a* with high level of similarity with tilapia *ostf1* [4]. Meanwhile, both gene expression was shown to be upregulated under hyperosmotic stress. Moreover, ostf1b modulates the expression of aquaporin 1 (AQP1), cystic fibrosis transmembrane conductance regulator (CFTR), and sodium hydrogen antiporter 3 (NHE3) for osmoregulation [4]. OSTF1 acts as a multifunctional transcription factor in fish. It participates in different downstream functions in different species, such as osmoregulation in tilapia [1], medaka [4], and Japanese eel (*Anguilla japonica*) [3], embryogenesis in zebrafish (*Danio rerio*) [5], and sex change in orange-spotted grouper (*Epinephelus coioides*) [6].

According to bioinformatics analysis, OSTF1 is a transcription factor characterized by a leucine zipper domain. It is a member of the transforming growth factor-beta stimulated clone 22 (tsc22) family. OSTF1 is also known as transforming growth factor-beta stimulated clone 22 domain 3 (tsc22d3) or glucocorticoid-induced leucine zipper (GILZ) [1,2,7]. In mammals, GILZ has been found to be an anti-inflammatory regulator that binds to proinflammatory transcription factors (e.g., nuclear factor kappa light chain enhancer of activated B cells, NF-κB) [8,9]. In addition, GILZ in mammalian kidney epithelial cells interacts with several epithelial Na^+^ channel (ENaC) regulatory proteins (e.g., serum- and glucocorticoid-induced kinase 1, SGK1) to regulate the expression and activity of ENaC by aldosterone through the MAPK pathway [10].

Cortisol, a glucocorticoid stress hormone, enables seawater (SW) adaptation in fish [11]. Cortisol affects the development and morphology of ionocytes in fish gills. Therefore, it is involved in increasing the ability of fish to osmoregulate in SW [12–14] and in upregulating several hyperosmotic stress-responsive genes [15–19]. On the other hand, cortisol plays a critical role in the energy substrate reallocation, which is crucial for SW adaptation of fish [18]. Moreover, McGuire et al. (2010) reported upregulated *ostf1* expression in fresh water (FW)- or SW-acclimated tilapia gills after cortisol injection [20].

Milkfish (*Chanos chanos*) is a marine euryhaline teleost distributed in tropical and subtropical regions of the world [21–23]. Milkfish is one of the most important aquaculture species in Southeast Asian countries, including Indonesia, Philippines, and Taiwan [21,24,25]. It has outstanding osmoregulation ability and can survive in environments with salinity ranging from 0 to 150‰ [26,27].

To date, most studies on fish OSTF1 have examined FW inhabitant euryhaline species (e.g. tilapia and medaka) or catadromous species (e.g. eel), revealing that the gene expression level of *ostf1* increased after transfer to SW from FW [1,3,4]. There was only one study on a marine inhabitant euryhaline teleost, the black porgy (*Acanthopagrus schlegeli*), showing that the gene expression level of *ostf1* increased after transfer to FW from SW [28]. However, little is known about the expression of OSTF1 in marine euryhaline fish after exposure to hypoosmotic or hyperosmotic environments, and the effect of cortisol, the SW-acclimation hormone, on OSTF1 expression in marine euryhaline teleosts. To better understand the potential roles and regulatory mechanisms of OSTF1 in marine euryhaline teleosts, the present study aimed to investigate (i) the expression of OSTF1 in the gills of milkfish, a marine euryhaline species, when exposed to hypoosmotic (FW) or hyperosmotic (SW) environments, and (ii) the effects of cortisol on branchial OSTF1 expression in euryhaline milkfish.

## Materials and methods

### Experimental fish and environments

Juvenile milkfish (*Chanos chanos*) with 17.09 ± 1.50 g of weight and 10.55 ± 0.35 cm of length were obtained from the DaShun fish farm in Tainan, Taiwan. Milkfish were reared in brackish water (BW; 15‰) at 28 ± 1°C for two weeks, then transferred to either fresh water (FW) or seawater (SW) at 28 ± 1°C for four-weeks acclimation. BW and SW were prepared from local tap water containing appropriate amounts of artificial sea salt (Blue Treasure Tropic Fish Sea Salt, Qingdao, China). The photoperiod in the fish room was 12 h light:12 h dark. Water was continuously circulated through fabric-floss filters, and a quarter of the water was changed every two weeks. The fish were fed commercial milkfish pellet diets once daily.

### Acclimation, transfer, and cortisol injection experiments

For long-term acclimation experiments, milkfish were acclimated to FW or SW for at least four weeks before tissue sampling. For the transfer experiments, FW- and SW-acclimated milkfish were transferred from FW to SW or SW to FW. For the cortisol injection experiments, FW-acclimated milkfish were injected with cortisol or DMSO (control group). Fish were sampled at 0, 3, 6, 12, and 24 h after transfer or injection.

### Ethical statement

All experiments were conducted according to the principles and procedures approved by the Institutional Animal Care and Use Committee (IACUC) of National Chung Hsing University (IACUC approval no. 108–137 granted to T.H. Lee).

### Blood collecting and tissue sampling

The milkfish were fasted for one day and anesthetized with 0.05% (500 μL/L) 2-phenoxyethanol (PANREAC, Barcelona, Spain) before blood collection and sampling. Milkfish blood was collected from the caudal vein using a Li-heparinized syringe with 27 G needles. The plasma was collected after centrifugation (5,000 *× g* for 10 min at 4°C) of the blood and stored at -20°C before analysis. After blood collection, the milkfish were sacrificed, and all tissues were sampled. The tissues were immediately frozen in liquid nitrogen and stored at -80°C before homogenization.

### Total RNA extraction and reverse transcription (RT)

Total RNA was extracted using IsoI-RNA Lysis Reagent (5 Prime, Gaithersburg, MD, USA) following the manufacturer’s instructions. The RNA pellet was dissolved in sterilized distilled water and deionized water. The intact extracted RNA was verified using 1% agarose gel (SeaKem® LE Agarose; Lonza, Basel, Switzerland) electrophoresis with SafeView^TM^ Classic nucleic acid stain (ABM, San Jose, CA, USA). The concentration and quality of the extracted RNA were measured using NanoDrop 2000 (Thermo Fisher Scientific, Wilmington, DE, USA). Purified RNA with an A260/A280 ratio between 1.8 and 2.0, and an A260/A230 ratio over 2.0, were used for all RNA experiments. For RT, first-strand cDNA was synthesized from 1 μg of total RNA samples using the iScript^TM^ cDNA synthesis kit (Bio-Rad, Hercules, CA, USA) following the manufacturer’s instructions.

### Real-time PCR (RT-PCR) analysis

For RT-PCR, the primers for *osft1a* (XM_030764688.1), *ostf1b* (XM_030764689.1), and *β-actin* (XM_030789841.1) were designed using Primer 3 Plus (http://www.bioinformatics.nl/cgi-bin/primer3plus/primer3plus.cgi). The amplification efficiency from the standard curve of each primer pair used for RT-PCR analysis was within the range of 95–105% (Table 1). To analyze the gene expression of *osft1*, the MiniOpticon Real-Time PCR system (Bio-Rad) with SYBR green (2x KAPA SYBR^®^ FAST qPCR Master Mix; Kapa Biosystems, Wilmington, MA, USA) was used. All data were normalized using *β-actin* as the reference gene. To quantify gene expression level in this experiment, the comparative Ct value method was used with the following formula: 2^- [(Ct _target_, n–Ct _β-actin_, n)—(Ct _target_, control–Ct _β-actin_, control)]. In the formula, ‘Ct’ represented the threshold cycle number in the formula and ‘n’ represented each cDNA sample used in these experiments [29].

**Table 1:** Primer sequences used for real-time PCR in this study.

Genes	Primer	sequence 5’ to 3’	Amplicon size (bp)	Efficiency (%)
*Ostf1a*	CC_ostf1a_F544	CCATCGGACTGGACTGCT	189	96.8
	CC_ostf1a_R733	TCCAGGGAGTCCTGTCTCAT		
*Ostf1b*	CC_ostf1b_F161	CTTCAGCAGGCTGTTCTCG	180	96.6
	CC_ostf1b_F341	CTTGACAACAGTGCCTCTGG		
*β-actin*	CC_*β*-actin_F	CCATTGAGCACGGTATTGTCA	82	103.4
	CC_*β*-actin_R	GCAACACGCAGCTCGTTGTA		

F, forward strand; R, reverse strand.

### Cortisol injection and measurement of plasma cortisol concentration

Cortisol doses used in this study were chosen according to Hu et al. (2019) [30]. Hydrocortisone (Sigma) was dissolved in dimethyl sulfoxide (DMSO; Sigma) [11,16,30], and used for intraperitoneal injection. Milkfish were injected with DMSO and 4 μg cortisol/g body weight injection, respectively [30]. The volume of dissolved cortisol was calculated according to the average milkfish weight. Before injection, fish were anaesthetized by 0.05% (500 μL/L) 2-phenoxyethanol.

### Determination of cortisol concentration

Plasma was used to detect cortisol concentrations, which were determined using a fish cortisol ELISA kit (Cusabio Biotech, Wuhan, China) following the manufacturer’s instructions.

### Statistical analyses

In this study, the prediction of protein motifs of the milkfish OSTF1 sequences was analyzed by Myhits (https://myhits.isb-sib.ch/cgi-bin/motif_scan). The normality test was verified to test the distribution by the method of Shapiro-Wilk normality test, and nonparametric methods were used for data analysis. The tissue distribution and the comparison between cortisol and DMSO injection were analyzed using the Mann–Whitney test, and time-course data were analyzed using the Kruskal–Wallis test with Dunn’s multiple comparisons test. All analyses were performed using GraphPad Prism 8 (GraphPad Software, San Diego, CA, USA). Values are expressed as mean ± standard error of the mean (SEM), p<0.05 was set as the significance level.

## Results

### Classification of OSTF1 in milkfish

**Fig 1:**
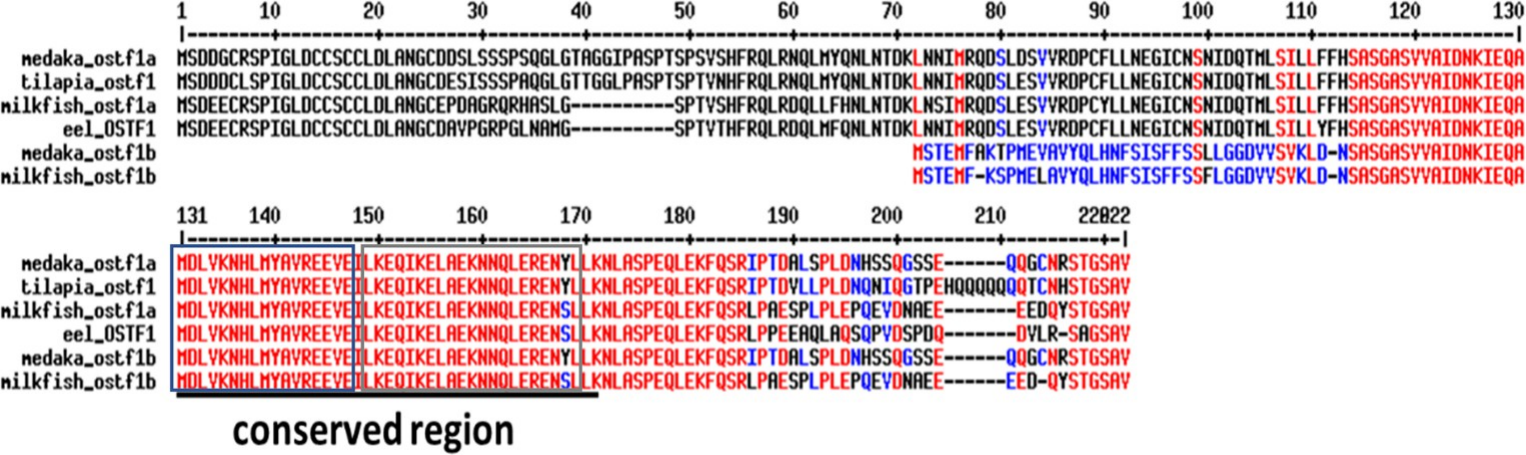
The amino acid sequence of milkfish OSTF1a and OSTF1b was aligned with OSTF1 sequences of other teleostean species. The identical amino acids were indicated by red residues, the low consensus amino acids were indicated by blue residues, and black residues represented the non-identical amino acids. The underline indicated the conserved region of OSTF1. The blue rectangle indicated the TSC22 domain, and the gray rectangle indicated the leucine zipper.

The milkfish *ostf1* sequences, *tsc22d3×1* (XM_030764688.1) and *tsc22d3×2* (XM_030764689.1) (S1 Fig), were searched in the NCBI database. Based on the amino acid sequences similarities between milkfish TSC22D3 and medaka OSTF1a and OSTF1b, these two sequences were named OSTF1a (TSC22D3×1) and OSTF1b (TSC22D3×2), respectively. By alignment of the amino acid sequences of milkfish OSTF1 with those of the other teleosts (Fig 1), milkfish OSTF1a revealed more than 80% similarity with the sequences of the tilapia OSTF1, eel OSTF1, and medaka OSTF1a. The sequence similarity of OSTF1b was also found between milkfish and medaka. The two milkfish OSTF1 sequences shared almost 100% similarity with other teleosts in the conserved region. Based on the motif prediction, both OSTF1 sequences of milkfish have the TSC22 domain and leucine zipper (Fig 1).

### Tissue distribution of *ostf1a* and *ostf1b* in FW- and SW-acclimated milkfish

**Fig 2:**
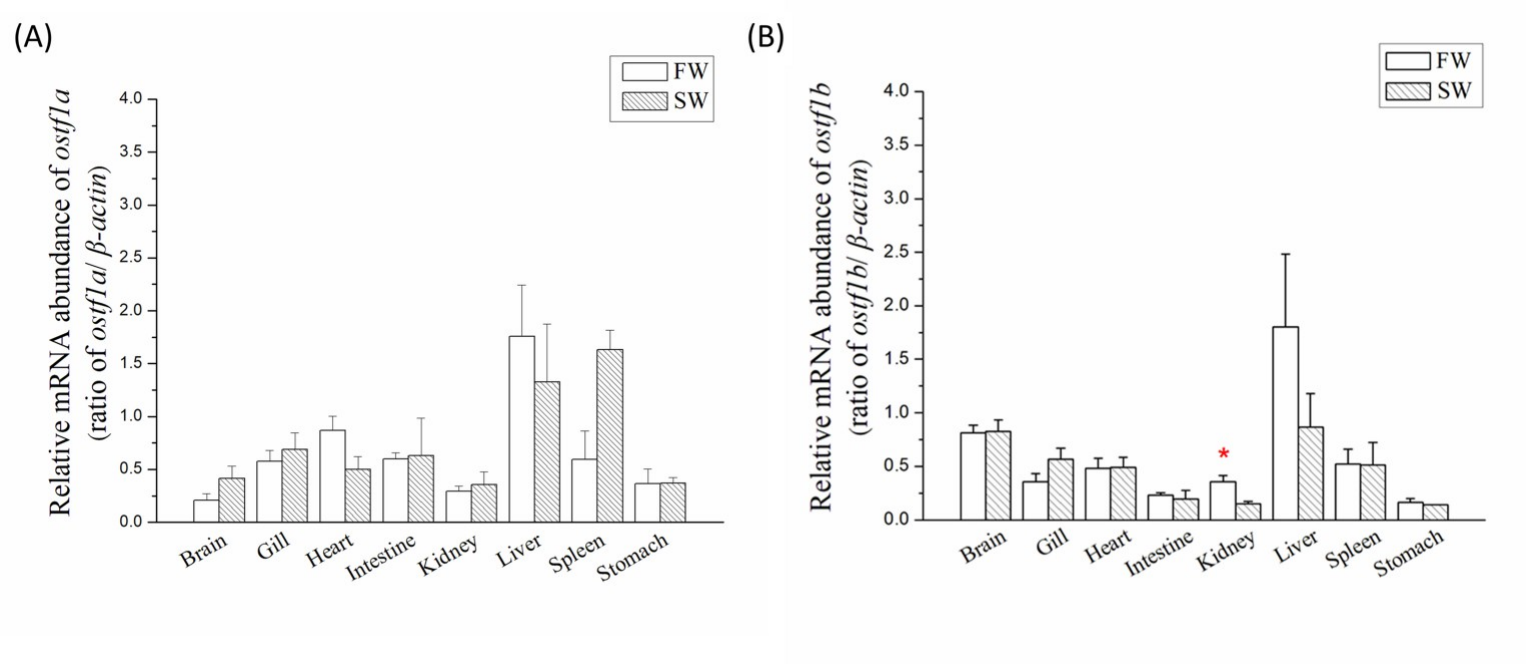
Tissue distribution of *ostf1* mRNA abundance in milkfish acclimated to fresh water or seawater. (A) *ostf1a* and (B) *ostf1b* was detected by real-time PCR. The expression has been normalized according to the expression of *β-actin*. Values are shown in means ± S.E.M. Gill and kidney, n = 6; other tissues, n = 3 (p < 0.05, analyzed by Mann–Whitney test). The asterisks indicated significant differences between the FW and SW group.

In FW- and SW-acclimated milkfish, *ostf1a* and *ostf1b* were expressed in all the tissues (Fig 2). Moreover, *ostf1a* was mainly expressed in the liver of FW- and SW-acclimated milkfish and in the spleen of SW-acclimated milkfish (Fig 2A), whereas the highest abundance of *ostf1b* was found in the liver of milkfish (Fig 2B). Meanwhile, *ostf1a* and *ostf1b* were expressed in all detected targets. Significant differences in *ostf1a* and *ostf1b* levels were found in the spleens and kidneys, respectively, between the FW and SW groups. However, in the gills, the expression levels of *ostf1a* (Fig 2A) and *ostf1b* (Fig 2B) were similar in FW- and SW-acclimated milkfish.

### Gene expression of *ostf1a* and *ostf1b* in gills of milkfish upon salinity challenges

**Fig 3:**
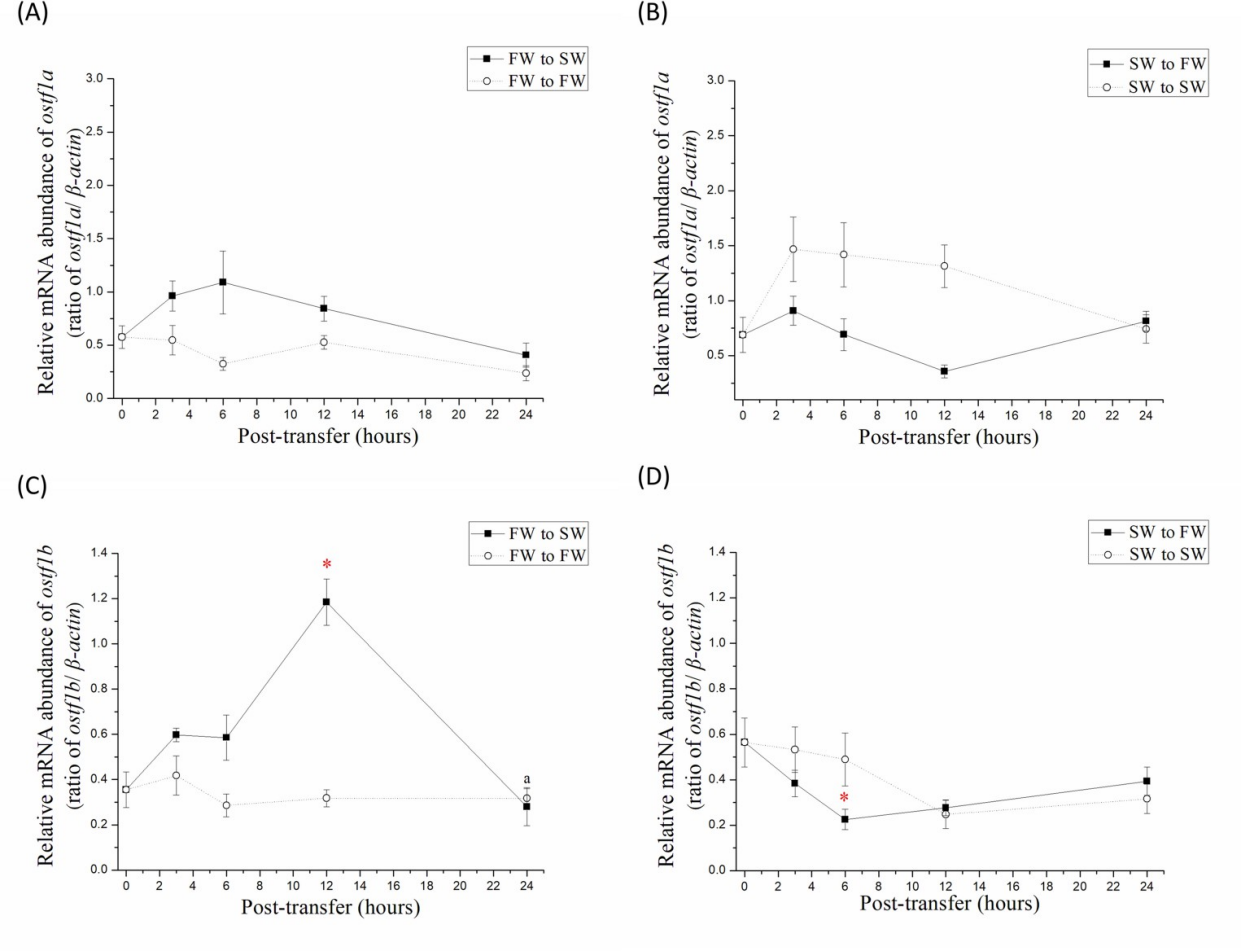
Time-course mRNA abundance of *ostf1* in gills of milkfish transferred from FW to SW and SW to FW for 24 h. (A, B) *ostf1a* and (C, D) *ostf1b* was detected by real-time PCR. The expression has been normalized according to the expression of *β-actin*. The time-points of sampling are 0, 3, 6, 12 and 24 h, respectively. Values were shown in means ± S.E.M. (n = 6). The asterisks indicated significant differences between each time-points and 0 h (p < 0.05, analyzed by Kruskal–Wallis test with Dunn’s multiple comparisons test).

To determine whether *ostf1a* and *ostf1b* are rapid response genes to hyperosmotic or hypoosmotic stress in the gills of milkfish, one-month FW- and SW-acclimated milkfish were directly transferred to SW and FW, respectively, and set as the salinity-transfer (study) groups. Meanwhile, FW- and SW-acclimated milkfish directly transferred to FW and SW, respectively, were set as the control groups. In the SW-transfer study group, the expression level of gill *ostf1a* did not change significantly after transfer (Fig 3A). However, the gene expression of gill *ostf1b* in the SW transfer group increased significantly at 12 h post-transfer and decreased back to the original level 24 h after transfer (Fig 3C). On the other hand, in the FW-transfer study group, the expression of the *ostf1a* in gills was similar (Fig 3B), while branchial *ostf1b* expression was significantly lower at 6 h post-transfer from SW (0 h) (Fig 3D).

### Plasma cortisol concentration of milkfish upon salinity challenges

**Fig 4:**
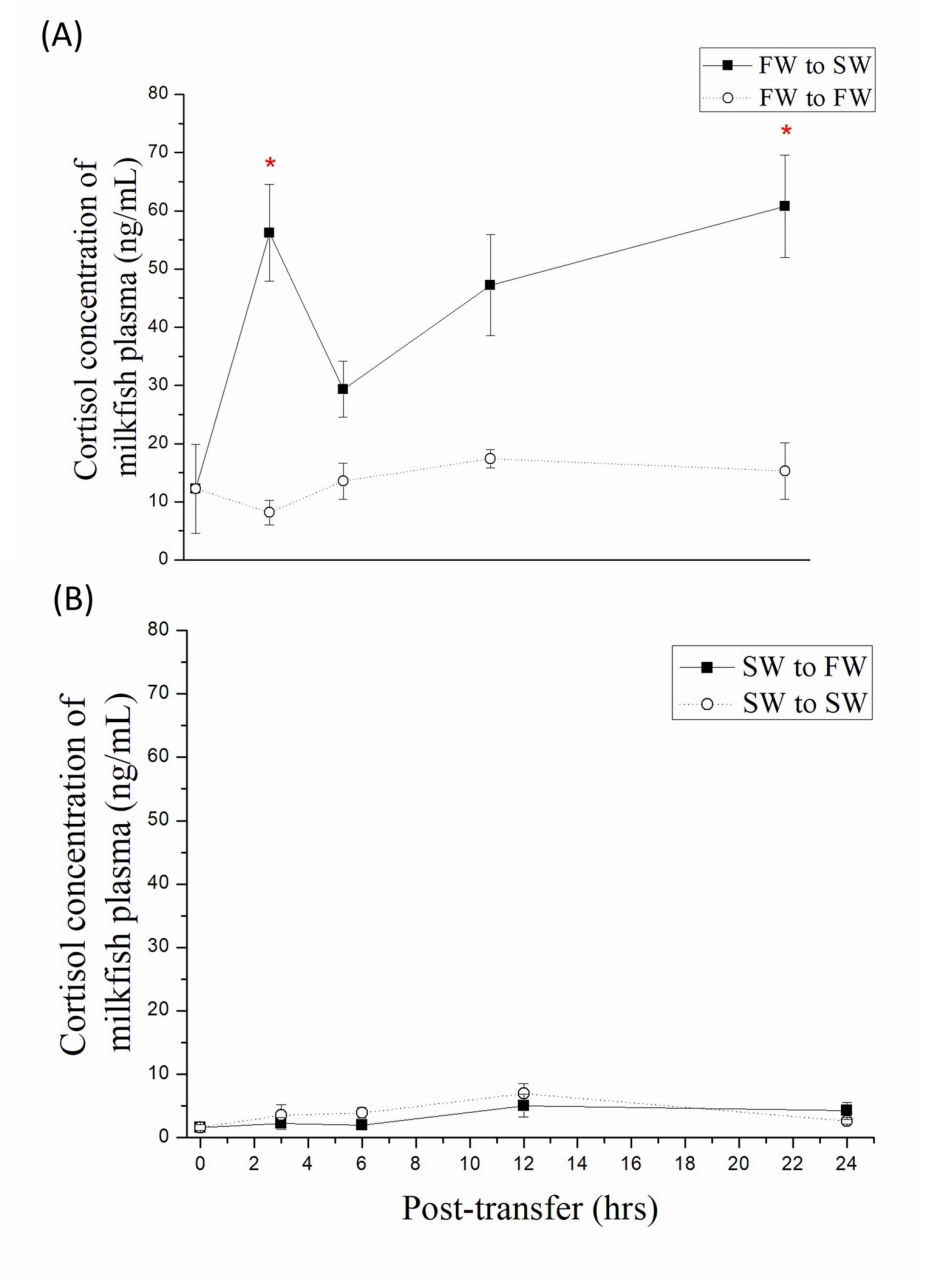
Plasma cortisol concentration of milkfish transferred from (A) FW to SW and (B) SW to FW for 24 h. Values were shown in mean ± S.E.M. (n = 6). The asterisks indicated significant differences between each time-point and 0 h (p < 0.05, analyzed by Kruskal–Wallis test with Dunn’s multiple comparisons test).

To determine the profile of cortisol concentration after salinity transfer in milkfish blood, the study and control groups of SW-transfer and FW-transfer milkfish were performed as previously described. After transfer, milkfish plasma was collected at each time point. The cortisol concentration in the plasma of the SW-transfer study group increased rapidly and significantly at 3 and 24 h post-transfer compared to FW (0 h) (Fig 4A). In contrast, plasma cortisol levels were steady in both the study and control groups of the FW transfer milkfish at 24 h post-transfer from SW (Fig 4B).

### Gene expression of *ostf1a* and *ostf1b* in gills of milkfish after cortisol injection

**Fig 5:**
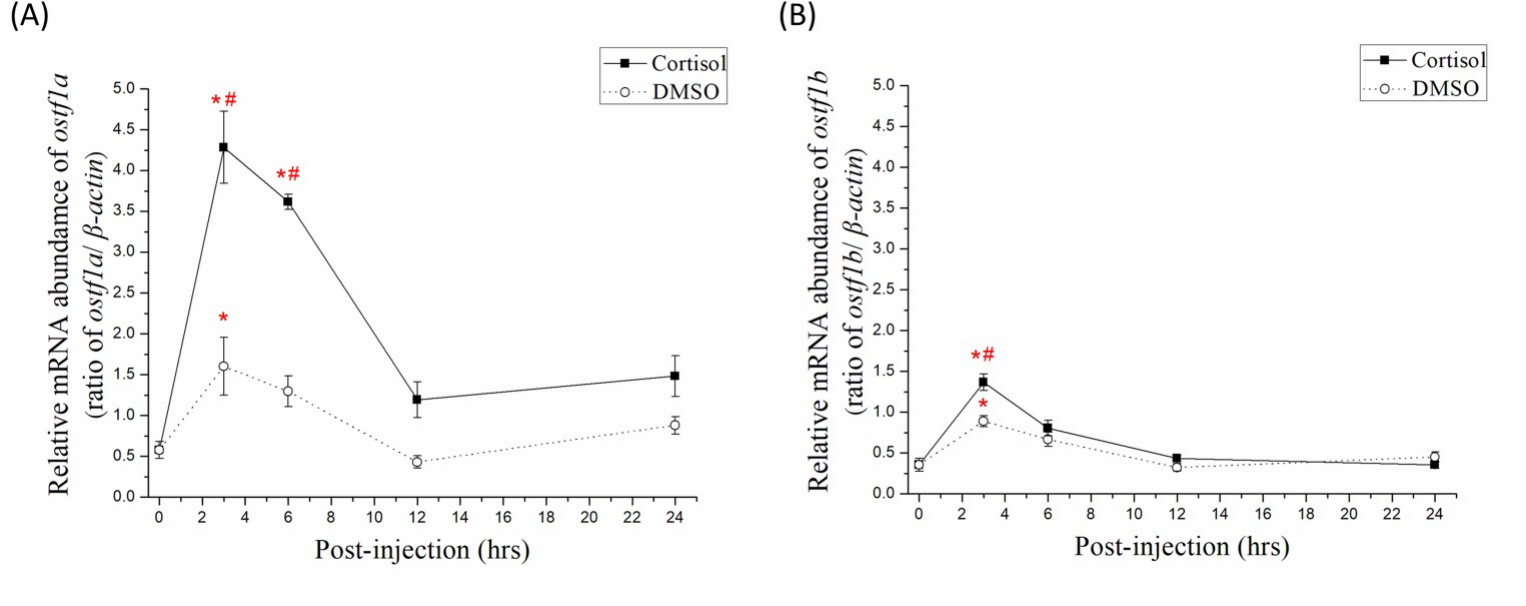
Time-course mRNA abundance of (A) *ostf1a* and (B) *ostf1b* in gills of FW-acclimated milkfish after injection with cortisol and DMSO for 24 h. The abundance has been normalized according to the expression of *β-actin*. Values were shown in mean ± S.E.M. Treatment group, n = 6; control group, n = 4. The asterisks indicated significant differences between each time-point and 0 h (p < 0.05, analyzed by Kruskal–Wallis test with Dunn’s multiple comparisons test). The sharps indicated significant differences between the cortisol group and DMSO group at the same time-point (p < 0.05, analyzed by Mann–Whitney test).

To illustrate the relationship between *ostf1* and cortisol, plasma cortisol concentration in FW milkfish was measured after cortisol injection (S2 Fig), and ostf1a and ostf1b expression was detected. Comparisons between different time-points of the cortisol groups at 0 h showed that the gene expression level of *ostf1a* in gills of the FW milkfish increased significantly at 3 and 6 h (7–9 folds on average) and returned to the background level at 12 h after cortisol injection (Fig 5A). The gene expression level of *ostf1b* increased significantly at 3 h (4 folds on average) and returned to the background level after 6 h (Fig 5B). In the DMSO group, *ostf1a* and *ostf1b* expression levels increased significantly (3–4 folds on average) 3 h after injection. Comparisons between the cortisol and DMSO groups at the same time points showed that the gene expression level of *ostf1a* was significantly higher at 3 and 6 h (Fig 5A), while that of *ostf1b* was significantly higher at 3 h (Fig 5B).

## Discussion

OSTF1 was first identified in the gills of Mozambique tilapia and as a transcription factor with a leucine zipper domain [1]. Subsequently, studies on *ostf1* in medaka gills were conducted on its expression and possible participation in the JNK pathway [4]. In medaka, two *tsc22d3* transcripts have been named *ostf1a* and *ostf1b* [4]. This study revealed that in the seawater (SW) inhabitant milkfish, two *tsc22d3* transcripts were found in the NCBI database. Based on the similarity of amino acid sequences between tilapia and medaka, the milkfish TSC22D3 was renamed as OSTF1a or OSTF1b.

Most fish studies focused on the expression and functions of *ostf1* in gills [1,3,4,28]. There is limited understanding of *ostf1* expression in other tissues. To our knowledge, this study is the first to reveal the tissue distribution of *ostf1* in fish. In milkfish, both *ostf1a* and *ostf1b* are expressed in all tissues. The *ostf1a* was highly expressed in the liver and spleen of SW-acclimated milkfish, whereas *ostf1b* was highly expressed in the liver, and significantly higher in the kidney of FW milkfish. The gill and kidney are important organs for osmoregulation, and the liver and spleen are crucial for immune functions in teleosts [31,32]. In Atlantic salmon (*Salmo salar*), microarray analysis showed that the gene expression levels of *ostf1* decreased in the liver, muscle, and head kidney after bacterial injection [33]. In mammals, *tsc22d3* is expressed in different organs and tissues, and it participates in adipogenesis, immunity, and renal sodium transport [34]. In zebrafish, *ostf1* may participate in embryonic development [5]. Taken together, *ostf1* in fish may be a multifunctional transcription factor for maintaining osmoregulation, as well as playing other physiological roles.

In the gills of freshwater (FW) euryhaline fish, e.g., tilapia, medaka, and eel, *ostf1* was found to be a rapid response gene to salinity challenge. The gene expression level of *ostf1*(b) increased 4–10 folds in 2–6 h after transfer from FW to SW, but later returned to the background level [1–4]. In the gills of milkfish transferred from FW to SW, the expression level of *ostf1b* elevated and reached its highest level at 12 h, and then returned to the background level at 24 h. Therefore, similar expression levels of branchial *ostf1b* were found in FW- and SW-acclimated milkfish. On the other hand, the expression level of *ostf1b* decreased significantly after 6 h of transfer from SW to FW. Accordingly, branchial *ostf1b* expression in marine euryhaline milkfish was found to respond rapidly when exposed to hypoosmotic environments, although it responded later than medaka, tilapia, and eels upon hyperosmotic challenge. Moreover, *ostf1a* expression in medaka gills increased significantly after SW exposure [4], whereas *ostf1a* expression in milkfish gills did not change significantly after exposure to hyperosmotic SW. Hence, milkfish *ostf1b* might be the major osmotic challenge, while milkfish *ostf1a* might be responsible for other physiological roles. In addition, when exposed to hyperosmotic stress, *ostf1* of marine euryhaline milkfish showed similar responses to other FW euryhaline teleosts.

Cortisol plays an important role in the SW adaption of euryhaline teleosts [17–19]. Cortisol is critical for SW tolerance maintenance in the larval stage of the summer flounder (*Paralichthys dentatus*) [11]. In Asian sea bass (*Lates calcarifer*), cortisol can improve tolerance and survival in hypersaline environments, but not in hyposaline environments [35]. In long-term FW- and SW-acclimated milkfish, plasma cortisol levels were higher in FW than in SW milkfish [30]. In tilapia, the plasma cortisol levels were similar between FW and SW after one-month acclimation [36]. However, the plasma cortisol concentration in tilapia increases rapidly when exposed to hyperosmotic environments [37]. As in tilapia, this study revealed that after transfer from FW to SW, the plasma cortisol concentration of milkfish increased immediately. However, cortisol concentration was stable when milkfish were transferred from SW to FW. Furthermore, compared with Hu et al. (2019) [30], cortisol concentration might decrease after the transfer of milkfish from FW to SW in one day. Accordingly, in marine euryhaline milkfish, cortisol was found to be an important hormone in response to acute salinity challenge; in this regard, the marine euryhaline milkfish was similar to other FW euryhaline teleosts.

McGurie et al. (2010) reported that *ostf1* expression was induced by cortisol in Mozambique tilapia [20]. In this study, after intraperitoneal injection of cortisol, two *ostf1* genes identified in milkfish reached their highest expression levels at 3 h, related to changes in plasma cortisol levels after cortisol injection. As a result, cortisol can upregulate the gene expression of milkfish *ostf1a* and *ostf1b*. In addition, the increase in *ostf1a* level was greater than that in *ostf1b* level. Meanwhile, *ostf1a* was not found to be the primary gene when milkfish were exposed to hyperosmotic stress. We assumed that cortisol was not the primary factor in activating milkfish *ostf1b* in response to salinity stress, and that other factors contributed to *ostf1b* upregulation but did not increase *ostf1a* expression in response to salinity challenge. Furthermore, after injection with DMSO (the control group), the gene expression levels of *ostf1a* and *ostf1b* also increased in a short time but were 2–3 folds lower than those in the cortisol-treated group. Therefore, it will be intriguing to clarify whether *ostf1b* responds to hypertonic stress in milkfish gills and whether *ostf1a* responds to other unknown functions. In the present study, cortisol upregulated the gene expression of *ostf1b* in marine euryhaline milkfish, similar to freshwater euryhaline tilapia [20].

## Conclusion

In summary, the two milkfish TSC22D3 sequences were classified as OSTF1a and OSTF1b. Both *ostf1a* and *ostf1b* were expressed in all examined tissues and were most abundant in the liver, but not in the gill or other osmoregulatory organs. In milkfish gills, however, only *ostf1b* expression was salinity-dependent upon osmotic stress. In addition, cortisol levels were enhanced after milkfish were transferred from FW to SW, and gene expression levels of both *ostf1a* and *ostf1b* increased after cortisol treatment. Taken together, we assumed that other factors, but not cortisol, would activate milkfish ostf1b in response to hyperosmotic challenge. In addition to osmoregulation, *ostf1a* and *ostf1b* may play other roles in physiological homeostasis maintenance. More research is needed to understand underlying functions of OSTF1 and the relationship between cortisol and OSTF1 in milkfish.

## Supporting information

S1 Fig The nucleotide sequence alignment of milkfish OSTF1a and OSTF1b. The identical amino acids were indicated by red residues, the low consensus amino acids were indicated by blue residues, and black residues represented the non-identical amino acids.
(TIF)

S2 Fig Plasma cortisol concentration in FW-acclimated milkfish after injection with cortisol (treatment group) or DMSO (control group) for 24 h. Values were mean ± S.E.M.. Treatment group, n = 6; control group, n = 4. The asterisks indicated significant differences between each time-point and 0 h (p < 0.05, analyzed by Kruskal–Wallis test with Dunn’s multiple comparisons test).
(TIF)
