# Supplementary figures and images for: Rapid response of osmotic stress transcription factor 1 (OSTF1) expression to salinity challenge in gills of marine euryhaline milkfish (Chanos chanos)
Source: PLoS One. 2022 Jul 6;17(7):e0271029. doi: 10.1371/journal.pone.0271029 (PMC9258805; doi:10.1371/journal.pone.0271029)

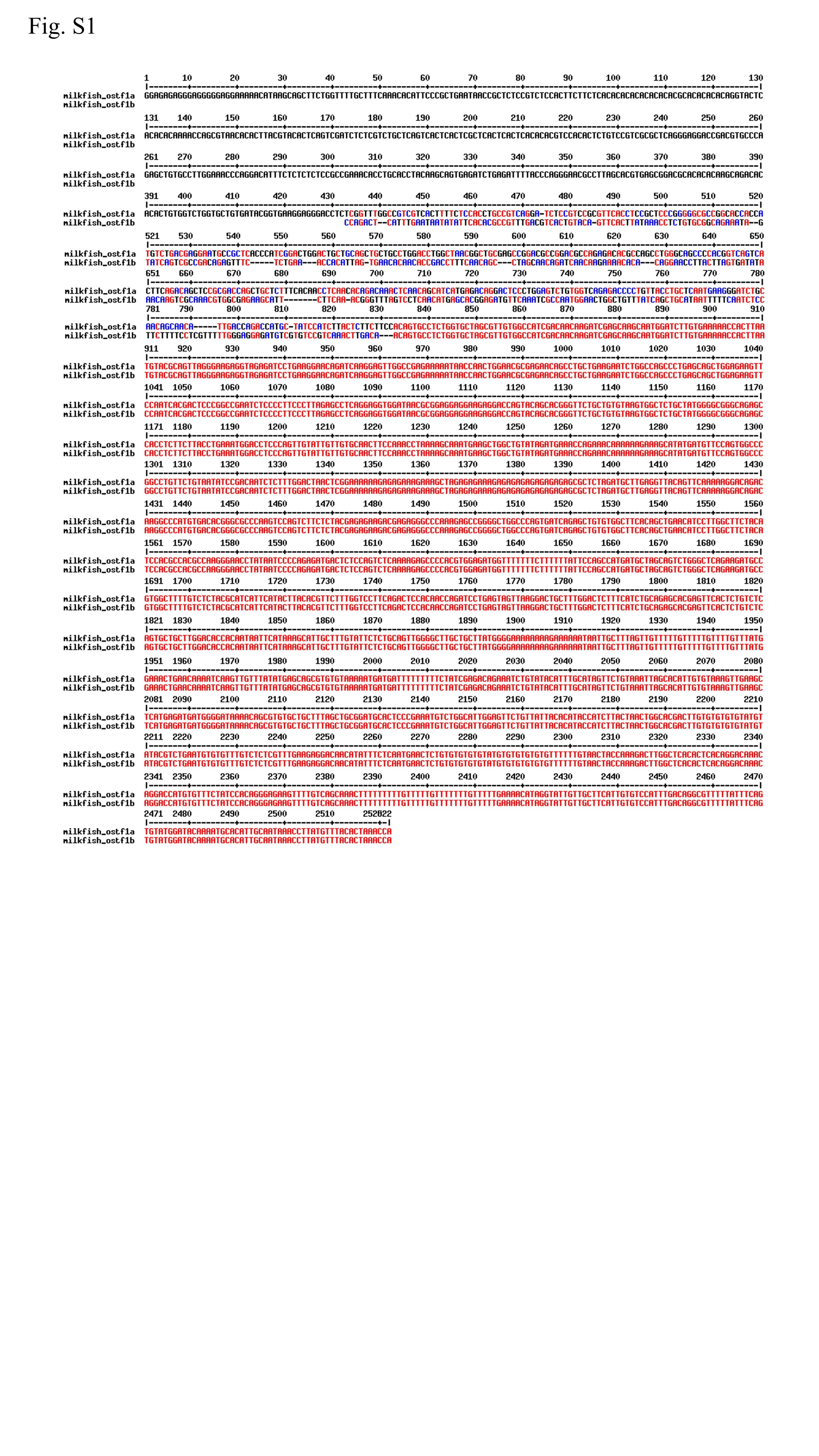

Supplement: S1 Fig — The identical amino acids were indicated by red residues, the low consensus amino acids were indicated by blue residues, and black residues represented the non-identical amino acids. (TIF) [file pone.0271029.s001.tif]

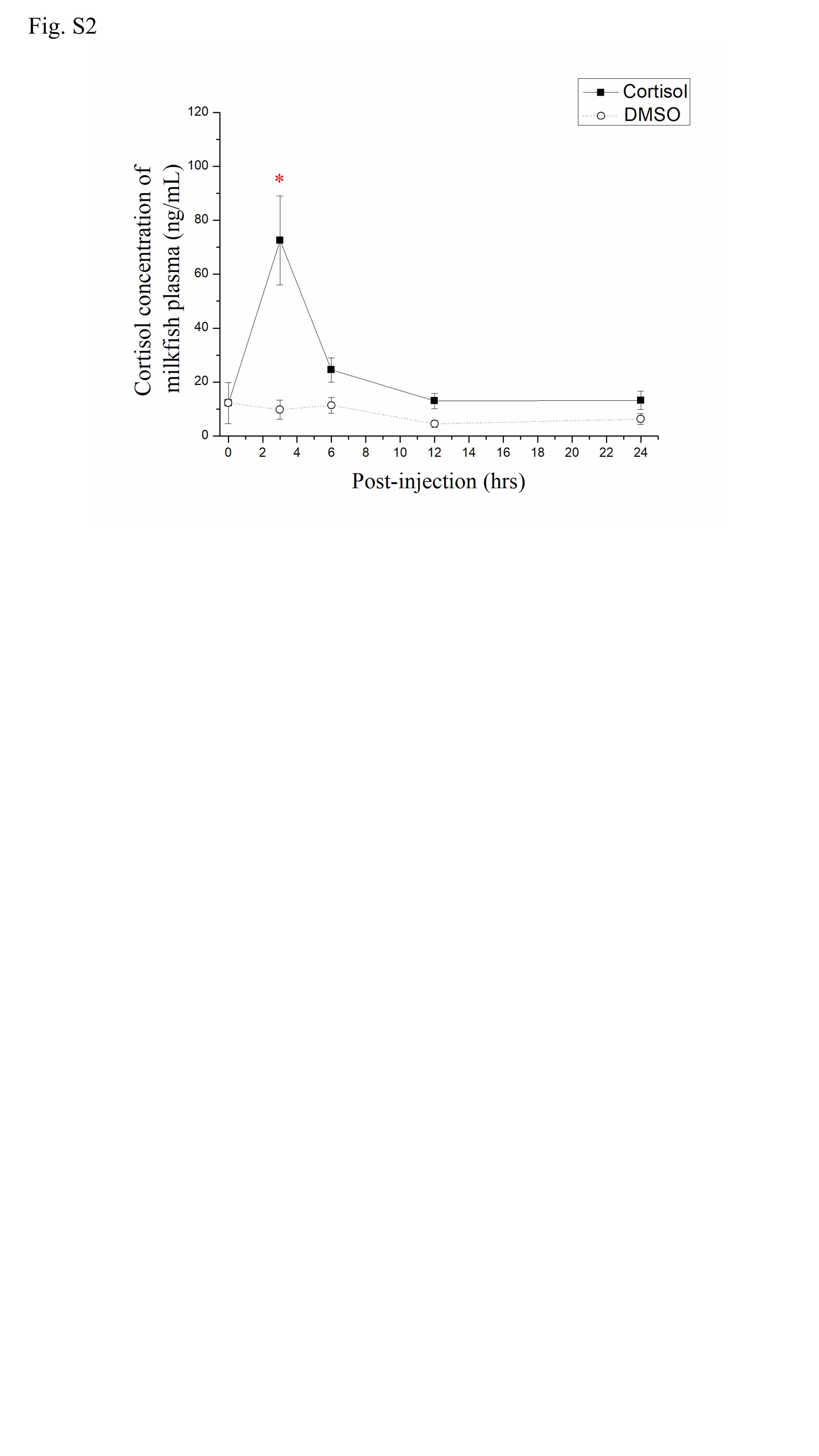

Supplement: S2 Fig — Values were mean ± S.E.M.. Treatment group, n = 6; control group, n = 4. The asterisks indicated significant differences between each time-point and 0 h (p < 0.05, analyzed by Kruskal–Wallis test with Dunn’s multiple comparisons test). (TIF) [file pone.0271029.s002.tif]
